# Effects of Jia-Wei-Xiao-Yao-San on the Peripheral and Lymphatic Pharmacokinetics of Paclitaxel in Rats

**DOI:** 10.1155/2016/5614747

**Published:** 2016-03-08

**Authors:** Mei-Ling Hou, Chia-Ming Lu, Tung-Hu Tsai

**Affiliations:** ^1^Institute of Traditional Medicine, National Yang-Ming University, No. 155, Section 2, Li-Nong Street, Taipei 112, Taiwan; ^2^Graduate Institute of Acupuncture Science, China Medical University, No. 91, Hsueh-Shih Road, Taichung 404, Taiwan; ^3^School of Pharmacy, College of Pharmacy, Kaohsiung Medical University, No. 100, Shih-Chuan 1st Road, Kaohsiung 807, Taiwan; ^4^Department of Education and Research, Taipei City Hospital, No. 145, Zhengzhou Road, Datong District, Taipei 103, Taiwan

## Abstract

Paclitaxel is effective against breast cancer. The herbal medicine, Jia-Wei-Xiao-Yao-San (JWXYS), is the most frequent prescription used to relieve the symptoms of breast cancer treatments. The aim of the study was to investigate the herb-drug interaction effects of a herbal medicine on the distribution of paclitaxel to lymph. A validated ultraperformance liquid chromatography with tandem mass spectrometry (UPLC-MS/MS) method was used to determine the paclitaxel levels in rat plasma and lymph after intravenous infusion of paclitaxel alone with or without 7 days of JWXYS pretreatment. The pharmacokinetic results indicate that paclitaxel concentrations in plasma exceeded those in lymph by approximately 3.6-fold. The biodistribution of paclitaxel from plasma to lymph was 39 ± 5%; however, this increased to 45 ± 4% with JWXYS pretreatment. With JWXYS pretreatment, the AUC and *C*_max_ of paclitaxel in plasma were significantly reduced by approximately 1.5-fold, compared to paclitaxel alone. Additionally, JWXYS decreased the AUC and *C*_max_ of paclitaxel in lymph. However, the lymph absorption rate of paclitaxel with or without JWXYS pretreatment was not significantly changed (27 ± 3 and 30 ± 2%, resp.). Our findings demonstrate that when paclitaxel is prescribed concurrently with herbal medicine, monitoring of the blood pharmacokinetics of paclitaxel is recommended.

## 1. Introduction

Cancer is a global health problem. Surgery, chemotherapy, and radiotherapy are still the major conventional cancer therapies [1]. Paclitaxel is a natural product used as a chemotherapeutic agent against various malignant tumors including ovarian, breast, and non-small-cell lung cancers. The antitumor activity of paclitaxel is attributed to microtubule stabilization [2]. Although chemotherapy and radiotherapy are effective treatments against cancer, these treatments can cause serious side effects and complications, including fatigue, pain, diarrhea, nausea, vomiting, and hair loss, that cannot be ignored [1, 3, 4]. Thus, increasing evidence demonstrates that herbal medicines can be used as adjuvant therapies to ameliorate the chemotherapy-induced side effects [5–7].

According to a survey from the National Health Insurance (NHI) database in Taiwan, Traditional Chinese Medicine (TCM) is frequently prescribed to people with cancer as a remedy for alleviating symptoms or improving quality of life [8]. The results demonstrate that the Chinese herbal formula Jia-Wei-Xiao-Yao-San (JWXYS) is the most frequently prescribed formula for treating breast cancer [8, 9]. Generally, JWXYS relieves climacteric symptoms including panic, dysphoria, and hot flashes in postmenopausal women [6]. Recently, JWXYS has been prescribed as an alternative treatment for chronic diseases [6, 8, 9].

Paclitaxel is a P-glycoprotein (P-gp) substrate and is metabolized via cytochrome P450 (CYP) 2C8 and the 3A4 subfamily [10]. Coadministration of drugs that modulate the activity of CYP enzymes is likely to have undesirable clinical consequences [5, 11, 12]. The study suggested that oral administration of some phenolic substances might increase paclitaxel blood concentration during chemotherapy [13]. Various clinical factors including lymph node invasion and tumor size are essential in determining the best therapeutic option for breast cancer patients [3, 4]. Differences in pharmacokinetics, and drug distribution in particular, may cause poor efficacy of the chemotherapeutic drug at the targeted site [3, 4]. The pharmacokinetics of paclitaxel in rats and humans has been investigated [14–16]; however, information regarding the distribution of paclitaxel in lymph remains limited. In addition, JWXYS is the most frequent prescription used to relieve symptoms in women being treated for breast cancer [8]. It is possible that JWXYS and paclitaxel may interact to exhibit adverse effects in the clinic. Thus, the aims of the study are to investigate the pharmacokinetics of paclitaxel by UPLC-MS/MS in rats following intravenous infusion of paclitaxel and to explore whether pretreatment with JWXYS affects the pharmacokinetics and lymph distribution of paclitaxel. These results provide information crucial to conducting chemotherapy in combination with JWXYS.

## 2. Materials and Methods

### 2.1. Chemicals and Reagents

The chemicals paclitaxel, docetaxel, and* tert*-Butyl methyl ether (TBME) were purchased from Sigma-Aldrich Chemicals (St. Louis, MO, USA). LC/MS grade solvents were obtained from J.T. Baker, Inc. (Phillipsburg, NJ, USA), and chromatographic reagents were obtained from Tedia Co., Inc. (Fairfield, OH, USA). Sodium chloride was purchased from E. Merck (Darmstadt, Germany). Triple deionized water (Millipore, Bedford, MA, USA) was used for all preparations. The pharmaceutical herbal product JWXYS manufactured in accordance with Good Manufacturing Practice (GMP) for Chinese Crude Drugs was obtained from pharmaceutical companies in Taiwan and has been used medicinally for patients. JWXYS consists of roots of* Angelica sinensis* (Oliv.) Diels, rhizomes of* Atractylodes macrocephala* Koidz., roots of* Paeonia lactiflora* Pall., roots of* Bupleurum chinensis* DC or* Bupleurum scorzonerifolium* Willd., sclerotia of the parasitic plant* Poria cocos* (Schw.) Wolf, roots and rhizomes of* Glycyrrhiza uralensis* Fisch., root barks of* Paeonia suffruticosa* Andr., ripe fruits of* Gardenia jasminoides* Ellis, rhizomes of* Zingiber officinale* Rosc., and stems and leaves of* Mentha haplocalyx* Briq., with a weight ratio of 4 : 4 : 4 : 4 : 4 : 2 : 2.5 : 2.5 : 4 : 2, respectively [17]. The pharmaceutical herbal product, JWXYS, was purchased from Sheng Chang Pharmaceutical Co., Ltd. (Taipei, Taiwan).

### 2.2. UPLC-MS/MS

The UPLC-MS/MS analysis was performed using a Waters Acquity UPLC*™* system (Waters, Manchester, UK) consisting of a binary solvent manager, an automatic liquid chromatographic sampler, and a Waters Xevo*™* tandem quadrupole mass spectrometer equipped with an electrospray ionization (ESI) source. Separation was achieved using a Phenomenex Kinetex C18 analytical column (100 × 4.6 mm, length × inner diameter, particle size of 2.6 *μ*m) maintained at 40°C in a column oven. The mobile phase consisted of triple deionized water and methanol (28 : 72; v/v) and the flow rate was set at 0.35 mL/min. The injection volume was 5 *μ*L.

For operation in the MS/MS mode, the electrospray ion source was operated with polarity positive ion mode in a single run. The ESI parameters were set as follows: source temperature: 150°C; desolvation temperature: 550°C; desolvation gas flow: 1000 L/h. The optimized cone voltages (CV) were 58 V for paclitaxel and 56 V for docetaxel. The multiple reaction monitoring (MRM) mode using specific precursor/product ion transitions was used for quantification. The molecular ions of paclitaxel and docetaxel were fragmented at collision energies of 28 and 26 eV using argon as the collision gas. Ion detection was performed by monitoring the transitions:* m/z *876.4 → 308.1 for paclitaxel and* m/z *830.4 → 549.2 for docetaxel. Docetaxel was used as the internal standard (IS) for positive ion mode analytes. A MassLynx 4.1 software data platform was used for spectral acquisition, spectral presentation, and peak quantification.

### 2.3. Animal Experiments

All animal experimental protocols were reviewed and approved by the Institutional Animal Care and Use Committee (IACUC number: 1020716) of National Yang-Ming University. Male specific-pathogen-free Sprague-Dawley rats weighing 300 ± 20 g were obtained from the Laboratory Animal Center of the National Yang-Ming University, Taipei, Taiwan. Animals were provided free access to food (laboratory rodent diet 5P14, PMI Feeds, Richmond, IN) and water.

In accordance with published studies, the doses of paclitaxel and JWXYS for the animals were based on the human doses and were derived using the following conversion equation recommended by the US Food and Drug Administration guidelines: human equivalent dose (HED, mg/kg) = animal dose (mg/kg) × (animal *K*_*m*_/human *K*_*m*_) [18]. Clinically, paclitaxel is given as intravenous infusion for at least three hours, and the maximum daily dose of JWXYS for human is 12 g.

To investigate the herb-drug interaction effects of JWXYS on the pharmacokinetics of paclitaxel in rat plasma and lymph, paclitaxel was administered by intravenous infusion to rats with or without oral JWXYS at 1.23 g/kg for 7 days. The commercial pharmaceutical powdered JWXYS was weighed and suspended in sterile water at the dosing volume of 10 mL/kg. JWXYS at 1.23 g/kg was administered to each rat by oral gavage. At the 7th dose of JWXYS, rats were given 1 mL of olive oil by oral gavage thirty minutes preoperatively and then anesthetized with urethane (1 g/kg, IP) for mesenteric lymph duct, jugular vein, and femoral vein cannulation. The purpose of the oral dosing of olive oil 30 mins before operation was to facilitate identification of the lymph vessels [19, 20]. The procedure for the cannulation of the mesenteric lymph vessels was performed as previously described [21, 22]. The rats were administered 5 mg/kg of paclitaxel by intravenous infusion into the right femoral vein at the infusion rate of 2 mL/hour for 3 hours. Two hundred *μ*L samples of blood were withdrawn from the cannula implanted in the jugular vein into heparin-rinsed vials at 0, 15, 30, 60, 120, 180, 185, 195, 210, 225, 240, 270, 300, 330, 360, 390, and 420 min. The lymph was collected into heparin-rinsed vials at 30 min intervals. The mesenteric lymph rate during the 24 h period following surgery averaged 2.4 ± 1.1 mL/h [19]. In order to compensate the loss of body fluid, the rats were intravenously infused with normal saline at an infusion rate of 2 mL/hour throughout the experiment according to the published literature [19, 20]. The plasma samples were immediately centrifuged at 6000 rpm for 10 min.

### 2.4. Sample Preparation

The biological samples were prepared by liquid-liquid extraction. The plasma and lymph samples (90 *μ*L, resp.) spiked with 10 *μ*L of docetaxel solution (IS, 2.5 *μ*g/mL) were extracted by TBME for liquid-liquid extraction. Briefly, biological samples were extracted using 1 mL of TBME twice, vortexed for 5 min, and centrifuged at 13,000 rpm for 10 min. After centrifugation, the upper organic layer containing the paclitaxel was transferred to a new tube and evaporated to dryness using a vacuum pump. The dried residue was reconstituted with 100 *μ*L of mobile phase (triple deionized water : methanol = 28 : 72) and analyzed by UPLC-MS/MS. If the paclitaxel concentration was in excess of 500 ng/mL, then the plasma and lymph samples were diluted by blank plasma and lymph samples at an appropriate ratio before analysis.

### 2.5. Preparation of Standard Samples

Stock solutions of paclitaxel and IS were prepared in methanol at a concentration of 1 mg/mL. The calibration curves were obtained from biological samples freshly spiked with IS and the stock solution of paclitaxel at concentration ranges of 5–500 ng/mL. The spiked biological samples were extracted following the sample preparation procedure described above.

### 2.6. Method Validation

The method validation assays for quantification of paclitaxel in rat plasma and lymphatic fluid were conducted based on the current US Food and Drug Administration (FDA) bioanalytical method validation guidelines [23]. Specificity, matrix effects, and recovery were evaluated. Biological samples were quantified using the ratio of the peak area of each analyte to that of the IS. Peak area ratios were plotted against analyte concentrations. All linear curves were required to have a coefficient of estimation of at least >0.995. Using the UPLC-MS/MS method described above, the intra- and interday variability were determined by quantitating six replicates at concentrations of 5, 10, 25, 50, 100, 250, and 500 ng/mL on the same day and on six consecutive days, respectively. The accuracy (% bias) and the relative standard deviation (RSD %) were calculated.

To evaluate matrix effect (ME) and recovery (RE), six different lots of blank plasma or lymph samples were extracted and then spiked with paclitaxel at three concentrations. The corresponding peak areas of paclitaxel in the spiked biological samples after extraction (A) were compared to those of the aqueous standards in mobile phase (B) at equivalent concentrations. The ratio (A/B × 100) is defined as the ME. The corresponding peak areas of paclitaxel in the spiked biological samples before extraction (C) were compared to those of paclitaxel in the spiked biological samples after extraction (A) at equivalent concentrations. The ratio (C/A × 100) is defined as the RE.

### 2.7. Pharmacokinetic Data Analysis

Pharmacokinetic calculations were performed on each individual data set by noncompartmental methods using WinNonlin Standard Edition, version 1.1 (Pharsight Corp., Mountain View, CA).

### 2.8. Statistical Analysis

Data were summarized as the mean ± SD or SEM. Comparison between two groups was performed using the unpaired Student's *t*-test. Statistical significance was set at *p* < 0.05.

## 3. Results

### 3.1. Optimization of the UPLC-MS/MS Method

**Figure 1:**
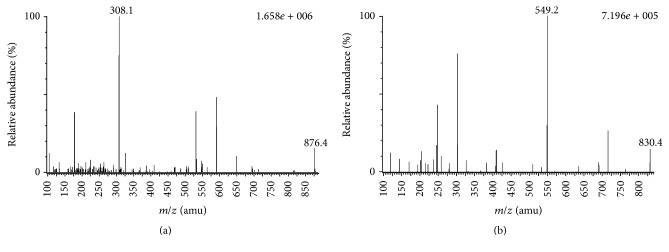
Mass spectra of (a) paclitaxel (*m/z *876.4 → 308.1) and (b) docetaxel (*m/z *830.4 → 549.2).

For parameter optimization, a standard solution of paclitaxel or docetaxel was analyzed by direct injection in the spectrometer. A full scan in the positive mode with precursor-product combinations monitored in the MRM mode was used for analyte identification. Paclitaxel and the IS could be ionized under positive (ESI^+^) electrospray ionization conditions. During initial infusion experiments with paclitaxel and docetaxel, the spectra revealed that both paclitaxel and docetaxel form Na^+^ adducts. Under ESI^+^ conditions, the precursor ions for paclitaxel and docetaxel were [paclitaxel + Na]^+^ at* m/z* 876.4 and [docetaxel + Na]^+^ at* m/z* 830.4, respectively. The MRM mode provided high selectivity and sensitivity for the quantification assay (Figure 1). The results of MS transitions demonstrate that the precursor ion of paclitaxel at* m/z* 876.4 [M + Na]^+^ and its main product ion at* m/z* 308.1 and the precursor ion of docetaxel at* m/z* 830.4 [M + Na]^+^ and its main product ion at* m/z* 549.2 were used to determine the analytes in the biological samples. Chromatographic conditions were optimized for good sensitivity, peak shape, and a relatively short run. Methanol provided the best peak shape and was selected as the organic phase. A mobile phase consisting of a water-methanol solution (isocratic elution) was used in the experiment.

### 3.2. UPLC-MS/MS Method Validation of Paclitaxel in Rat Plasma and Lymph

**Figure 2:**
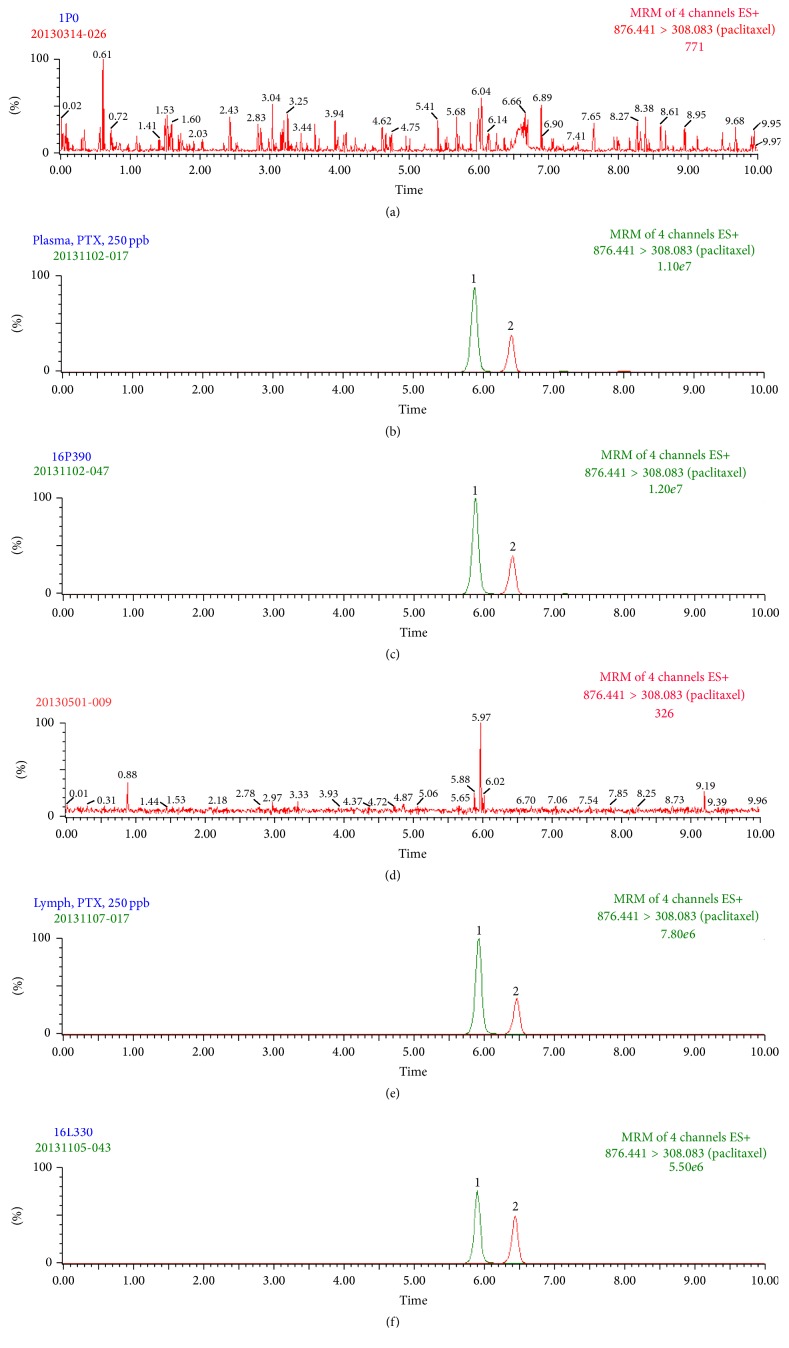
Representative chromatograms of paclitaxel in rat plasma and lymph. (a) Blank rat plasma; (b) blank rat plasma sample spiked with paclitaxel (250 ng/mL) and docetaxel (IS); (c) real rat plasma sample containing paclitaxel (270 ng/mL) collected at 390 min after administration of paclitaxel (5 mg/kg, i.v. infusion). (d) Blank rat lymph; (e) blank rat lymph sample spiked with paclitaxel (250 ng/mL) and docetaxel (IS); (f) real rat lymph sample containing paclitaxel (245 ng/mL) collected from 330 to 360 min after administration of paclitaxel (5 mg/kg, i.v. infusion). 1: paclitaxel; 2: docetaxel (IS, 250 ng/mL).

Assay specificity was assessed by comparing the chromatograms of blank plasma and lymph samples obtained from six rats with corresponding spiked plasma and lymph samples. Each blank plasma and lymph sample was tested using a liquid-liquid extraction procedure and UPLC-MS/MS conditions to ensure no interference of paclitaxel and IS from plasma and lymph. The results showed that no interference existed under the present analytical conditions (Figures 2(a) and 2(d)).

Figures 2(a) and 2(d) show the chromatograms of a blank plasma and lymph extract with mass transitions of* m/z *876.4 → 308.1 for paclitaxel and* m/z *830.4 → 549.2 for docetaxel (IS), illustrating a clean baseline with no interference peaks eluted within 10 min. Figures 2(b) and 2(e) show the chromatograms of a standard solution of paclitaxel (250 ng/mL) and IS (250 ng/mL) spiked in blank rat plasma and lymph.  Figure 2(c) shows the chromatograms for a plasma sample containing paclitaxel (270 ng/mL) collected at 390 min after dosing with paclitaxel (5 mg/kg, i.v. infusion).  Figure 2(f) shows the chromatograms for a lymph sample containing paclitaxel (245 ng/mL) collected from 330 to 360 min after dosing with paclitaxel (5 mg/kg, i.v. infusion). Each determination was completed within 10 min and no carry-over peaks were detected in the subsequent chromatograms of biological samples.

A 100% matrix effect value indicated that the response in the mobile phase and in the biological extracts was the same [20]. The mean matrix effects of paclitaxel and docetaxel (IS) in plasma were 106 ± 14 and 84 ± 5%, respectively; the mean matrix effects of paclitaxel and docetaxel (IS) in lymph were 80 ± 7 and 60 ± 5%, respectively (Table 1). The mean recovery for paclitaxel and IS in plasma was 85 ± 13 and 78 ± 4%, respectively; the mean recoveries for paclitaxel and IS in lymph were 84 ± 11 and 53 ± 5%, respectively (Table 1).

The calibration curve was constructed by plotting the peak area ratios of paclitaxel to the IS versus the concentrations of paclitaxel. The results demonstrated a linear response over the concentration ranges of 10–500 ng/mL, with a coefficient of estimation *r* ^2^ > 0.995. The data showed excellent reproducibility (Table 2). The limit of detection (LOD) and quantification (LOQ) were defined as the concentration of paclitaxel detected as a signal-to-noise (S/N) ratio of 3 and 10, respectively. The LOD and LOQ of paclitaxel in rat plasma and lymph were 5 ng/mL and the LOQ was 10 ng/mL. The intra- and interday precision (% RSD) and accuracy (% bias) values of paclitaxel in rat plasma and lymph were within 15% (Table 2). These results show that the UPLC-MS/MS method provides excellent quantitative analysis of paclitaxel in rat plasma and lymph extracts.

### 3.3. Herb-Drug Interaction Effects of JWXYS on the Blood Pharmacokinetics of Paclitaxel

**Figure 3:**
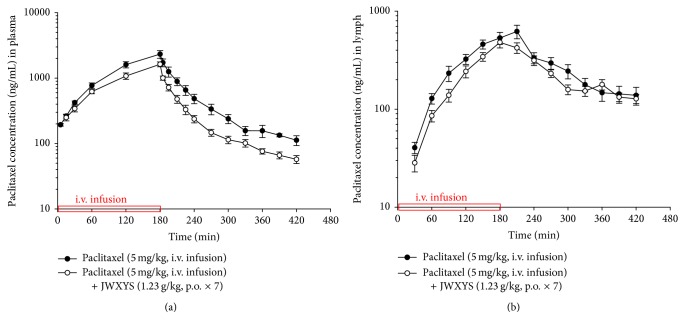
Mean concentration-time profiles of paclitaxel in rat plasma (a) and lymph (b) after intravenous infusion of paclitaxel (5 mg/kg) with or without JWXYS (1.23 g/kg, p.o. for 7 days) pretreatment. Each point represents mean ± SEM (*N* = 11).

The mean plasma concentration-time profiles of paclitaxel after intravenous infusion of paclitaxel at 5 mg/kg (*n* = 11) with or without pretreatment with JWXYS (1.23 g/kg, p.o. for 7 days) are illustrated in Figure 3(a) and the pharmacokinetic parameters are listed in Table 3. Following 7 days of pretreatment with oral JWXYS at 1.23 g/kg, the AUC and *C*_max_ of paclitaxel in plasma were significantly reduced by approximately 1.5-fold, compared to paclitaxel alone (Table 3). Additionally, the elimination half-life (*T*_1/2_) of paclitaxel in plasma was significantly prolonged 1.5-fold; the total body clearance (CL) increased by 43% and the volume of distribution (Vd) significantly increased 1.4-fold following coadministration with JWXYS. However, the mean residence time (MRT) exhibited no changes with or without pretreatment with JWXYS.

### 3.4. Herb-Drug Interaction Effects of JWXYS on the Lymph Pharmacokinetics of Paclitaxel

The mean concentration-time profiles of paclitaxel in rat lymph (*n* = 11) after intravenous infusion of paclitaxel (5 mg/kg) with or without pretreatment with JWXYS (1.23 g/kg, p.o. for 7 days) are illustrated in Figure 3(b), and the pharmacokinetic parameters are illustrated in Table 3. As shown in Figure 3, the drug concentration versus time curve of paclitaxel in rat plasma and lymph after intravenous infusion of paclitaxel with or without pretreatment with JWXYS indicated that the amount of paclitaxel in rat lymph was lower than the amount of paclitaxel in rat plasma. Paclitaxel concentrations in plasma exceeded those in lymph by approximately 2.8-fold, with AUC values of 315 ± 38.2 min *μ*g/mL in plasma and 113 ± 12.7 min *μ*g/mL in lymph. The *C*_max_ of paclitaxel in plasma was 3.6-fold greater than that in lymph.

Following 7 days of pretreatment with JWXYS, the AUC of paclitaxel in lymph was reduced by approximately 1.3-fold and the *C*_max_ yielded similar results (Table 3). However, the* T*_1/2_, Vd, CL, and MRT were not changed. The lymph absorption rate of paclitaxel (AUC_lymph_/AUC_plasma+lymph_ × 100) was 27 ± 3%, indicating that paclitaxel can be absorbed into the lymph system. However, pretreatment with JWXYS did not significantly affect the lymph absorption rate of paclitaxel (30 ± 2%) compared to paclitaxel alone. The biodistribution (AUC_lymph_/AUC_plasma_ × 100) of paclitaxel from plasma to lymph was 39 ± 5%; however, it increased to 45 ± 4% with JWXYS pretreatment. As shown in Figure 3, seven days of JWXYS at 1.23 g/kg significantly decreased the blood paclitaxel levels by approximately 30% and reduced the distribution of paclitaxel in rat lymph by around 24%.

## 4. Discussion

Although analytical methods have been reported for analysis of paclitaxel in biological matrices by high-performance liquid chromatography with UV [24, 25], and by tandem mass spectrometric detection [26, 27], there is no validated method for the determination of paclitaxel in rat lymph. The MS transitions of paclitaxel (*m/z *876.4 → 308.1) and docetaxel (*m/z *830.4 → 549.2) were in agreement with previous reports [27].

Numerous studies have indicated that the use of Chinese herbal medicines in combination with chemotherapy or radiotherapy for the treatment of cancer not only enhances the efficacy but also diminishes the side effects and complications induced by chemotherapy and radiotherapy treatments [5–9, 28]. Chinese herbal medicines, including Astragalus, Turmeric, Ginseng, TJ-41, PHY-906, Huachansu infection, and Kanglaite injection, are commonly used by patients for cancer treatment and/or for the reduction of toxicity associated with chemotherapy and radiotherapy [29]. Studies on pharmacokinetic interactions between Chinese herbal medicines and Western medicines [5, 11, 13, 30] have been conducted, and the results suggest that oral administration of some phenolic substances might increase paclitaxel blood concentrations during chemotherapy. Because paclitaxel is a P-gp substrate, investigation on P-gp inhibition on the mesenteric lymphatic transport of paclitaxel has been reported [31]. The results demonstrated that pretreatment with a P-gp inhibitor, verapamil, increased the lymphatic transport of paclitaxel by 3.5-fold and absolute oral bioavailability by 1.8-fold.

The bioactive components, including ferulic acid [32], atractylenolide I-III [33], paeoniflorin [34], saikosaponins [35], polysaccharides and triterpenoids [36], glycyrrhizin [37], paeonol [38], geniposide [39], zingerone [40], and chlorogenic acid [41], were found in the individual herb of JWXYS. Studies on herb-drug interactions in pharmacokinetics based on metabolizing enzymes have been reported. For example,* in vitro* and* in vivo* studies showed that the potential components of* Angelica sinensis* (Oliv.) Diels roots, one of the major herbs in JWXYS, have the inhibitory effects on CYP3A4 [42]. It is known that lactones of Artemisia (ligustilide), n-butene acid lactones, ferulic acid, nicotinic acid, sucrose, amino acids, and sesquiterpene compounds are pharmacologically active components of* Angelica sinensis* (Oliv.) Diels [32]. Additionally, licorice root of* Glycyrrhiza uralensis* Fisch. is another herbal component in JWXYS; its main active ingredient is glycyrrhizin [37].* In vitro* studies have shown that isoflavone glabridin isolated from* Glycyrrhiza uralensis* Fisch could have CYP3A4 enzyme induction properties [42]. Furthermore,* in vivo* studies showed that repeated treatment with glycyrrhizae or glycyrrhizic acid could increase CYP3A4 substrate metabolism, and CYP3A4 was induced at both the mRNA and protein levels [42].

Our pharmacokinetic results demonstrate decreased blood and lymph paclitaxel concentrations following 7 days of pretreatment with JWXYS. However, pretreatment with JWXYS did not significantly influence the distribution and absorption rate of paclitaxel in lymph. The results suggest that orally administered JWXYS affects metabolizing enzymes as well as disturbing the distribution of paclitaxel into the circulation* in vivo*. The possible mechanism of interaction between JWXYS and paclitaxel could be increased metabolism of paclitaxel or affect transporter functions in the intestinal lymphatic system following oral administration of JWXYS; consequently, this would decrease paclitaxel levels in the peripheral and lymphatic system.

The lymphatic system plays an important role in fluid/macromolecular balance, lipid absorption, and immune functions. It is involved in various pathologic conditions such as inflammation, spread of cancer cells, and lymphedema [43]. The lymphatic system can also transport dietary lipids, fat-soluble vitamins, and water-insoluble drugs to the systemic circulation. Paclitaxel has been approved to treat metastatic breast cancer [44]; however, limited evidence shows that paclitaxel can gain access to the lymphatic system. The only literature is the one in which Cai et al. has investigated the effects of lipid vehicle and P-gp inhibition on the mesenteric lymphatic transport of paclitaxel [31]. Few reports regarding the absorption of drugs into the lymphatic system [45–49] have been investigated due to practical difficulties in obtaining the lymphatic fluid. Studies on the determination of protein or water-insoluble compounds in lymph and plasma following intravenous or intestinal administration in rats have been reported [45–49]. The results suggest that, over a given dose range, a protein with a high molecular weight can be absorbed in both the portal vein blood stream and the thoracic duct lymph after intraduodenal administration of parotin [48]. Our pharmacokinetic results demonstrate that paclitaxel was detectable in rat lymph after intravenous infusion of paclitaxel; however, JWXYS significantly decreased blood paclitaxel levels by approximately 30%.

## 5. Conclusions

This is the first report to explore the herb-drug interaction effects of JWXYS on the pharmacokinetics of paclitaxel in rat peripheral and lymphatic systems. A validated UPLC-MS/MS method was applied for pharmacokinetic studies. The pharmacokinetic results demonstrate that pretreatment with JWXYS for 7 days significantly attenuated the distribution of paclitaxel in rat peripheral and lymphatic systems. Clinically, it is not practical to collect lymphatic fluid in patients following paclitaxel administration. These findings indicate that monitoring plasma levels of paclitaxel may help in assessing compliance and therapeutic effects and in dose optimization when coadministered with JWXYS.

## Figures and Tables

**Table 1:** Matrix effect and recovery of paclitaxel and docetaxel in rat plasma and lymph.

Nominal concentration (ng/mL)	Peak area			Matrix effect (%)	Recovery (%)
	Set 1	Set 2	Set 3		
*Plasma*					
Paclitaxel					
10	36872 ± 4813	32992 ± 2473	24279 ± 2632	89 ± 7	74 ± 8
100	235603 ± 12101	269808 ± 27215	237379 ± 31147	115 ± 12	88 ± 12
500	1202318 ± 61512	1356123 ± 74986	1265995 ± 133390	113 ± 6	93 ± 10
Average				106 ± 14	85 ± 13
Docetaxel (IS)					
250	465608 ± 9230	389823 ± 23305	302862 ± 16985	84 ± 5	78 ± 4
*Lymph*					
Paclitaxel					
10	30372 ± 3067	25645 ± 1935	20778 ± 2064	84 ± 6	81 ± 8
100	298923 ± 18866	246724 ± 5850	217499 ± 35420	83 ± 2	88 ± 14
500	2216455 ± 159883	1598223 ± 99812	1341908 ± 180573	72 ± 5	84 ± 11
Average				80 ± 7	84 ± 11
Docetaxel (IS)					
250	465609 ± 9330	280022 ± 21317	147762 ± 12713	60 ± 5	53 ± 5

Data expressed as mean ± SD (*N* = 6). Matrix effect is expressed as the ratio of the mean peak area of an analyte spiked after extraction (set 2) to the mean peak area of the same analyte standard (set 1) multiplied by 100. A value of >100% indicates ionization enhancement, and a value of <100% indicates ionization suppression. Recovery was calculated as the ratio of the mean peak area of an analyte spiked before extraction (set 3) to the mean peak area of an analyte spiked after extraction (set 2) multiplied by 100.

**Table 2:** Intra- and interday precision (% RSD) and accuracy (% bias) of the UPLC-MS/MS method for determination of paclitaxel in rat plasma and lymph (6 days, 6 replicates per day).

Nominal concentration (ng/mL)	Intraday			Interday		
	Observed concentration (ng/mL)	Precision (% RSD)	Accuracy (% bias)	Observed concentration (ng/mL)	Precision (% RSD)	Accuracy (% bias)
*Plasma*						
10	9.58 ± 1.28	13.4	−4.20	9.42 ± 1.36	14.4	−5.77
25	23.9 ± 1.22	5.13	−4.51	24.8 ± 2.11	8.50	−0.88
50	49.5 ± 2.90	5.87	−0.99	48.6 ± 3.25	6.69	−2.88
100	102 ± 7.27	7.10	2.45	99.1 ± 2.46	2.48	−0.89
250	259 ± 9.80	3.78	3.64	257 ± 7.19	2.80	2.76
500	495 ± 4.30	0.87	−1.05	497 ± 4.17	0.84	−0.62
*Lymph*						
10	9.65 ± 1.25	12.9	−3.49	9.21 ± 1.26	13.6	−7.88
25	25.1 ± 2.67	10.6	0.28	25.1 ± 2.34	9.34	0.25
50	52.2 ± 3.04	5.83	4.34	52.4 ± 3.47	6.62	4.70
100	101 ± 4.32	4.30	0.58	101 ± 3.44	3.39	1.42
250	247 ± 10.5	4.26	−1.32	248 ± 14.2	5.71	−0.76
500	502 ± 4.94	0.99	0.33	498 ± 7.66	1.54	−0.37

Data expressed as mean ± SD.

**Table 3:** Pharmacokinetic parameters of paclitaxel (5 mg/kg, i.v. infusion) with or without JWXYS (1.23 g/kg, p.o. for 7 days) pretreatment.

PK parameters	Paclitaxel (5 mg/kg, i.v. infusion)	Paclitaxel (5 mg/kg, i.v. infusion) + JWXYS (1.23 g/kg, p.o. × 7)
*Plasma*		
*C*_max_ (ng/mL)	2355 ± 314	1630 ± 141^*∗*^
AUC_0–420 min_ (min *µ*g/mL)	315 ± 38.2	209 ± 18.9^*∗*^
*T*_1/2_ (min)	73.7 ± 12.2	111 ± 12.8^*∗*^
Vd (L/kg)	1.39 ± 0.15	2.00 ± 0.17^*∗*^
CL (mL/min/kg)	17.5 ± 1.95	25.1 ± 2.55^*∗*^
MRT (min)	68.6 ± 3.70	62.9 ± 1.71
*Lymph*		
*C*_max_ (ng/mL)	648 ± 94.3	494 ± 58.7
AUC_0–420 min_ (min *µ*g/mL)	113 ± 12.7	89.8 ± 8.25
*T*_1/2_ (min)	116 ± 17.6	122 ± 7.0
Vd (L/kg)	8.08 ± 1.59	10.0 ± 1.09
CL (mL/min/kg)	45.7 ± 8.17	48.3 ± 4.36
MRT (min)	119 ± 4.77	130 ± 4.34
Biodistribution (%)	39 ± 5	45 ± 4
Lymph absorption rate (%)	27 ± 3	30 ± 2

Data expressed as mean ± SEM (*N* = 11). *C*_max_: the peak plasma concentration of a drug after administration; AUC: area under the concentration versus time curve; *T*_1/2_: elimination half-life; Vd: volume of distribution; CL: total body clearance; MRT: mean residence time; biodistribution, (AUC_lymph_/AUC_plasma_) × 100: the distribution of paclitaxel from plasma to lymph; lymph absorption rate, [(AUC_lymph_/AUC_plasma+lymph_) × 100]: the lymph absorption rate of paclitaxel. ^*∗*^Significantly different from paclitaxel alone at *p* < 0.05.
